# Medical tourism in ophthalmology - review

**DOI:** 10.22336/rjo.2022.22

**Published:** 2022

**Authors:** Consuela-Mădălina Gheorghe, Victor Lorin Purcărea, Iuliana-Raluca Gheorghe

**Affiliations:** * Department of Marketing and Medical Technology, “Carol Davila” University of Medicine and Pharmacy, Bucharest, Romania

**Keywords:** medical tourism, ophthalmology services, health care services

## Abstract

During the last decade, it seems that we have witnessed an upsurge in medical tourism and its documentation. Definitions often refer more to the terms themselves rather than to the medical tourists and frequently approach discussions using the principles of motivation, procedures, and tourism.

The aim of this review was to investigate the existing literature on medical tourism in health care services, and in Ophthalmology, and to assess whether the principles of medical tourism are successfully applied in health care services, with a specific interest in Ophthalmology services.

## Introduction

Literature on medical tourism and especially medical tourism in ophthalmology is scarce, the most common articles and research being the ones related to Dental Medicine, Cardiology, and Plastic Surgery services.

Medical tourism is an umbrella concept used to describe receiving medical care abroad and is defined as the act of leaving one’s own country in order to receive treatments and care overseas or domestically [**1**]. 

Since the internet has the particularity of connecting “customers” to “sellers” in healthcare-related services, online advertising emerged and contributed significantly to the development of various types of tourism. Consequently, the key motivators for medical tourism in ophthalmology are reflected in the availability of information about the treatment and surgeries. Additionally, decisions to purchase ophthalmological care abroad are influenced by the rising cost and the consumerism in the industrialized nations, but the subjective perceptions of the expenses must also be taken into consideration. The healthcare industry considers patient satisfaction as the key component of any medical system, which is based on high quality and innovation [**1**]. 

Moreover, it is argued that medical tourism or medical travel, as it is also referred to, has appeared in the late twentieth century [**2**]. However, not all the information about medical tourism is useful or reliable. 

Health tourism has hundreds of years of history but has been replaced, at present, by medical tourism, which has rapidly expanded in the last quarter of the century and deals with patients travelling abroad to health centers for medical treatment [**3**]. 

At the same time, medical tourism can be explained as the travel abroad with the aim of benefiting from non-emergency medical services [**4**]. 

## History

The current trend of medical travel dates to Antiquity everywhere in the world. Evidence suggests that various hill tribes from present-day Switzerland traveled to the former German and French colonies to rest at hot springs that, in their opinion, had therapeutic properties. The hot springs and Greek medical facilities were the first hubs dedicated to medical tourism. By that time, yoga and ayurvedic remedies were well established and were receiving a lot of attention in the Western world. The Industrial Revolution accelerated the introduction of new technology and advancements in the medical industry. By the end of the 20th century, the United States had become a major destination for patients traveling abroad for medical care. As the twenty-first century drew near, the medical tourism business become increasingly integrated and coordinated, and was recognized as an independent domain [**5**]. 

The growing trend of medical tourism has started in the late 1990s, when patients started to move to India, Thailand, and Mexico to benefit from cheaper health services than in their own countries. 

Medical tourism is considered an economic issue both at the system and individual levels. The decision of the patient to engage in medical tourism refers to the unsatisfied needs in terms of the type of the services being looked for and the way the treatment is applied [**6**]. 

Some authors argue that medical tourism is a form of globalization because its implications remain unknown, although it has both risks and benefits. Basically, it exists due to the high-care costs, long waiting periods or lack of access to new therapies in developed countries [**7**]. These are the motivations that convince people to travel to less developed countries for medical assistance.

What is clear is that the concept has an increasing relevance for the two fields, medicine, and tourism, as both use common concepts, as for instance, reputation and images to improve revenues and raise employment in the field [**8**]. 

Although medical tourism has been limited to cosmetic and plastic surgery and dental procedures in the last decades, it has recently expanded to almost all medical specialties, including ophthalmology. It seems that ophthalmology has the most advanced technology of all medical specialties in the world. A raise in demand has been registered for refractive procedures and oculofacial plastic surgery. Also, the countries that are often chosen as destinations for eye procedures are Turkey and Spain.

Romania is mainly known for the SPA like resources, and thermal baths. In addition to being a destination for medical tourism and a source for other medical destinations, primarily West European nations, Romania reflects European trends of increasing patient mobility in search of affordable medical care [**9**]. 

The Romanian Medical Tourism Association claims that the following procedures are appealing to foreign patients: dental work, cosmetic surgery, ophthalmology/ LASIK surgery, interventional cardiology, orthopedic surgery, other surgeries, CT and MRI scans, or SPA treatments for post-surgical recovery [**10**]. Bucharest and other important towns in the country, served by functional airports like Cluj-Napoca, Timișoara, Târgu Mureş, Sibiu, Constanța, are the most frequented cities for medical tourism [**11**]. 

As mentioned previously, medical tourism has similarities to and connections with the tourism sector. Diaspora travelers are only vaguely familiar with what is available back “home”, and depending on the seriousness of the surgery, many rely almost completely on word of mouth. Since the internet has taken over, most medical tourism guidebooks that were produced in the middle and late 2000s have not been updated. Although the internet has become essential for marketing, it only predominates in surveys of populations that are not necessarily planning to travel for medical purposes [**2**] or in regions where the medical tourism sector is just starting to emerge [**2**].

Most prospective patients visit the websites of hospitals and clinics before choosing one, and health providers frequently advertise online. The availability of procedures, dependability, quality, and cost are their main concerns; however, the last one is largely emphasized, except for Asia (where prices are cheaper), on the theory that most prospective patients have already learned about it. Technology-based images of modernity, cleanliness, and apparent efficiency predominate.

Most websites are in the English language and offer information on a variety of procedures, fees, accreditation and affiliations, knowledgeable personnel, opulent wards and accommodations, patient testimonials, and multilingual proficiency. In any type of marketing, technological progress is rarely disregarded [**2**]. 

## Advantages

Medical tourism has been highly influenced by advancements in technology and the quality of life. 

Moreover, ophthalmological treatments in Romania are an effective way to recover following a surgery, as they are more affordable than in other industrialized nations and offer a wide range of medical tourism amenities.

The advantages of medical tourism and clearly of medical tourism in ophthalmology are related to cost effectiveness, insurance incentives, luxury and private nursing, vacation in a foreign country and bypassing the rules and regulations.

Cost effectiveness represents the most common reason for seeking cheaper medical care. Regarding insurance incentives, due to observable savings, many insurance organizations have started to promote medical tourism. Only few patients, who are having medical treatments are attracted to the SPA-like centers and treatments that some remote hospitals offer because they can get an advantage and be cared without having to pay extra for travel, surgery, etc. People can combine medical and recreational travel, which is another important factor in medical tourism. Moreover, there are very few medical procedures that are not permitted in the countries where they are performed. Some patients choose to have their surgery performed abroad to avoid following local regulations set forth by the government, insurance company, or hospital [**5**]. 

## Disadvantages

Among the disadvantages of medical tourism are the medical, legal and the ethical risks. Risks associated with medical tourism exist and are not present when patients are treated in their homes. Patients frequently travel for medical treatment to countries with significantly different infectious disease epidemiologies than the ones they are from. Form a legal perspective, if a patient experiences a negative result from treatment as a result of a medical error, how would they be able to seek recourse, given the lack of internationally regulated rules pertaining to medical tourism? Numerous ethical dangers can be found in the workplace, when traveling, and afterwards during individual medical operations and rehabilitation. It is important to note that several brokers are engaged in the process of planning the trip and the treatment, and they frequently do not give customers enough information about the procedure and the potential consequences [**12**].

## Medical Tourism – a business opportunity

Medical tourism can be regarded as a good business opportunity from which the following stakeholders can benefit: medical organizations, patients, travel agencies, national governments, etc. 

The field of medical tourism is thought to be worth $100 billion. Very little is known about the primary forces behind medical tourism and how nations are viewed as medical tourism destinations, even though there are a rising number of people, businesses, and governments engaging in it [**13**]. 

During the period 2020-2025, the global medical tourism industry is anticipated to expand at a compound annual growth rate (CAGR) of 8.5 percent. The market is expected to rise as a result of reasons such as increasing healthcare costs in developed countries, new technology, high standards of care around the world, likelihood of having health insurance, and medical tourism advertising [**5**]. 

Since it serves as a hub for information exchange between potential patients and providers across continents, the Internet has had a significant impact on the industry’s growth [**14**]. 

## Rules for the patients who resort to medical tourism

When they decide to travel abroad, patients should consider some very important rules such as analyzing all the destinations available that treat the specific medical condition, trying to estimate the cost for consultation and possible surgery, in case it is needed, verifying the professionality of the doctor, verifying if the hospital or medical organization is accredited and has a good reputation, finding information regarding the cleanliness standards, being ensured from a medical point of view before leaving the native country, etc. However, there are also some rules regarding what the patients should not do, while on a medical treatment abroad: should refrain from doing physical exercises that involve physical force, should not leave the country without a consent from the caring doctor/ surgeon, should refrain from any physical activity and rest after any procedure, should not consider only the costs, should have a two-way communication with the doctor/ surgeon, should make reservations in advance, etc. 

To conclude, patients should make an appropriate planning and research when deciding to resort to medical tourism. 

## Reasons for choosing medical tourism in ophthalmology

Patients choose to travel for ophthalmological procedures because they can benefit from the latest technology, advanced treatments, best health care and medical services. 

Some of the most important destinations for medical tourism in ophthalmology are Turkey, India, Singapore, etc., and the most common procedures are cataract surgery, corneal transplant surgery, laser eye surgery, glaucoma surgery and treatment, artificial intraocular lens implantation, etc. 

## Conclusion

Since there is a great deal of variability, it is challenging to define medical tourism, and more in-depth research is needed for most medical tourism services categories. Even while some in the industry prefer the seriousness, accountability, and reliability connected with “medical travel” or “medical migration”, it is still a niche within tourism.

Strengthening the strategic marketing of medical tourism in ophthalmology and travel companies toward sustainable health tourism, it might significantly improve a country’s brand and help to prolong the seasonality of tourism activities.

Furthermore, several sociocultural impacts need to be analyzed, such as making health care less affordable for local patients and/ or moral and ethical concerns about certain treatment methods. Another area of concern, before traveling for medical tourism, relates to the legal issues of risk, liability, and malpractice.

**Conflict of Interest statement**

The authors state no conflict of interest.

**Acknowledgements**

None.

**Sources of Funding**

None.

**Disclosures**

None.
